# Gliosarcoma with Primary Skull Base Invasion

**DOI:** 10.1155/2016/1762195

**Published:** 2016-12-08

**Authors:** Quoc-Bao D. Nguyen, Avital Perry, Christopher S. Graffeo, Cody L. Nesvick, Aditya Raghunathan, Mark E. Jentoft, Brian P. O'Neill, Padraig P. Morris, Jonathan M. Morris, Jamie J. Van Gompel

**Affiliations:** ^1^Texas A&M Health Science Center College of Medicine, Temple, TX, USA; ^2^Department of Neurological Surgery, Mayo Clinic, Rochester, MN, USA; ^3^Department of Laboratory Medicine and Pathology, Mayo Clinic, Rochester, MN, USA; ^4^Department of Neurology, Mayo Clinic, Rochester, MN, USA; ^5^Department of Radiology, Mayo Clinic, Rochester, MN, USA; ^6^Department of Otolaryngology-Head and Neck Surgery, Mayo Clinic, Rochester, MN, USA

## Abstract

Gliosarcoma is an uncommon variant of glioblastoma, which commonly demonstrates dural attachment. However, skull base invasion is rarely seen with this entity. Herein, we report a 44-year-old female patient diagnosed with primary intracranial gliosarcoma extensively invading the skull base and muscles of mastication. She presented to our institution with a three-month history of difficult right jaw opening and retro-orbital pressure and one week of severe right-sided postauricular headache. Head CT demonstrated a 6 cm mass with marked bony erosion. Brain MRI at a one-week interval more clearly characterized tumor extension through the right orbit and muscles of mastication, with overall growth to 7 cm and worsening midline shift. The patient underwent a right frontotemporal craniotomy for gross total resection. Pathology confirmed the diagnosis of gliosarcoma, IDH-wildtype (WHO grade IV). Her postoperative course was uneventful and she was discharged at preoperative neurologic baseline. To our knowledge, this is the third reported case of a primary intracranial gliosarcoma with direct invasion of skull base, brain parenchyma, and extracranial compartment. However, this is the first report case of primary GS invading the surrounding musculature and orbit. This case report highlights the rapid aggressiveness of gliosarcomas and further a prior undescribed radiographic and anatomic finding of skull base invasion with this entity.

## 1. Introduction

Gliosarcoma (GS) is a rare variant of glioblastoma (GB), characterized by a biphasic tissue pattern, with alternating areas of glial and mesenchymal differentiation [1]. GS comprise 2–8% of all GB, are known to have a worse prognosis than GB, and have high prevalence in the 5th and 6th decades of life with a 2 : 1 male predilection—although isolated cases of congenital GS have been reported [1–5]. GS typically appear as rapidly growing, heterogeneously enhancing intra-axial masses comparable to GB with a temporal predominance [4, 6]. At resection, GS are observed as a firm, often times well-circumscribed, superficial lesion, with meningeal adhesions. GS invading the skull base with accompanying extracranial extension has not been previously documented in primary GS. We report the first case of primary intracranial GS with diffuse, multicompartment invasion of the surrounding skull base, brain parenchyma, orbit, and muscles of mastication, alongside a review of the relevant literature. This presentation would expand the radiographic differential in patients with lesions such as this.

## 2. Case Presentation

A 44-year-old woman presented with difficulty opening her right jaw, swelling in the right cheek and temple region, and right retro-orbital pressure. She had no history of radiation therapy or tobacco use. Family history was significant for multiple malignancies, including second- and third-degree relations with leukemia, prostate cancer, breast cancer, and ovarian cancer. She denied any family history of neurologic disease—including central nervous system neoplasms.

A presumed diagnosis of sinusitis was made, and a short course of steroids and antibiotics was initiated. Three months later, the patient's symptoms had not improved but rather had expanded to include severe headache and retroauricular pain with cervical radiation, as well as V2 distribution paresthesia. She presented to an outside emergency department, where head CT identified a 6 cm right temporal lobe mass growing through and destroying the greater wing of the sphenoid bone and invading into the infratemporal fossa. She was subsequently referred to our institution for further work-up and treatment.

**Figure 1:**
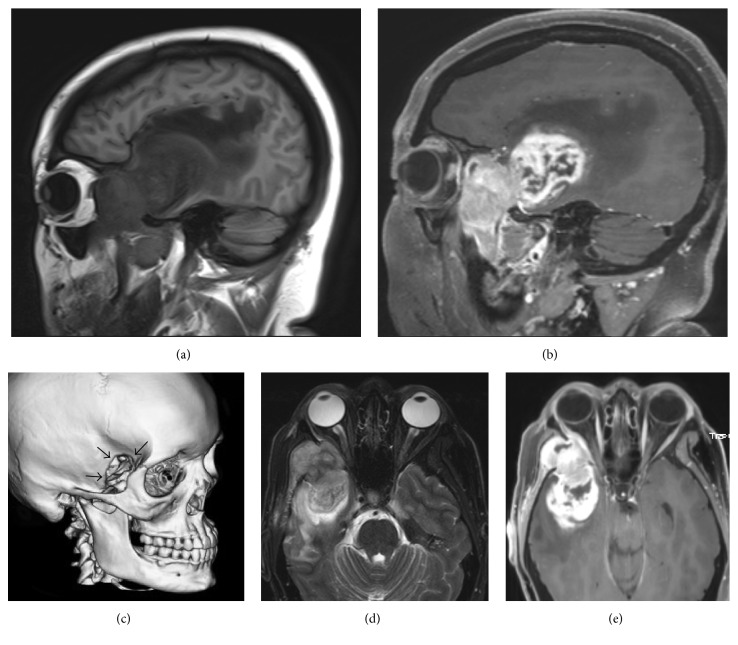
MRI and CT findings prior to surgery. (a) Sagittal T1-weighted imaging (T1WI) demonstrated a heterogeneous T1 hypointense mass involving a majority of the anterior temporal lobe with associated mass effect and surrounding vasogenic edema. Tumor extends through the greater wing of the sphenoid into the infratemporal fossa. (b) Sagittal postgadolinium T1WI demonstrates avid heterogeneous enhancement. The anterior portion in the soft tissues solidly enhances and intracranially has central necrosis. (c) 3D reconstruction from head CT demonstrates aggressive destruction of the greater wing of the sphenoid. (d) Axial FSE T2-weighted imaging demonstrates the mass to be heterogeneous iso/hypointense tumor with surrounding vasogenic edema suggesting increasing cellularity. (e) Axial postgadolinium T1WI demonstrates avid heterogeneous enhancement with central necrosis intracranially.

On examination, the patient was noted to have a firm right temporal mass, mild right proptosis, and right V2 paresthesias, but no other appreciable neurologic deficits. Brain MRI demonstrated marked growth, approximately 1 cm, over the one-week interval, with increased midline shift, mass effect, and vasogenic edema. The extracranial extension was better delineated with clear extension into the orbit, maxillary sinus, and invasion of the right pterygoid, masseter, and temporalis muscles (Figure 1). CT-guided biopsy of the temporal extracranial component was performed and histopathology revealed a tumor consistent with high-grade glioma versus GS, and an expedited operative resection was recommended.

**Figure 2:**
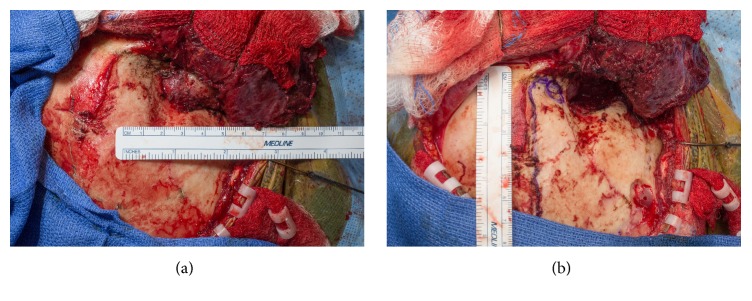
Intraoperative photos of the firm, encapsulated tumor penetrating through the temporal bone and involving the temporalis muscle, (a) before and (b) after debulking the tumor extracranially.

The patient was taken to the operating room for a right frontotemporal craniotomy, subtemporal exploration, tumor resection, and temporal lobectomy. During exposure, a firm and encapsulated tumor invasive through the temporal bone was encountered below the temporalis muscle (Figure 2(a)), which was debulked extracranially throughout infratemporal fossa and dissected off the periorbita, isolating the intracranial components (Figure 2(b)). After elevation of the frontotemporal bone flap and opening of the dura, the posterior tumor margin was identified and noted to be infiltrating adjacent cortex. An anterior temporal lobectomy with partial resection of the superior temporal gyrus and sparing of the mesial structures was completed to remove all intraparenchymal tumor and expose the portion invasive through the skull base at the inferomedial triangle between V1 and V2. The remaining bone was then drilled away and the lateral wall of the cavernous sinus was mobilized, exposing the lateral tumor margin and allowing for a gross total resection, including resection of all involved dura. The skull base was repaired in layers with pericranium, DuraGen, and abdominal fat graft.

**Figure 3:**
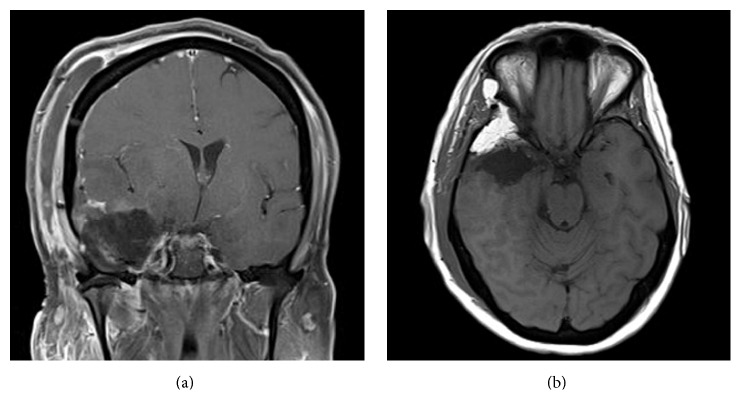
Postoperative T1-weighted gadolinium-enhanced MRI of the brain. Both (a) coronal and (b) axial sequences confirm gross total resection of all enhancing tumor. New hyperintensity appreciated at the anterior right temporal pole identifies abdominal fat graft placed during reconstruction.

The procedure was well tolerated and the patient recovered without complaints of jaw motion difficulties or new facial numbness. Routine MRI performed on postoperative day one revealed no evidence of residual enhancing tumor (Figure 3), and the patient was dismissed from the hospital on postoperative day three. Follow-up included close clinical and radiographic evaluations, as well as an adjuvant treatment plan of external beam radiation, 76 Gy in 30 fractions.

**Figure 4:**
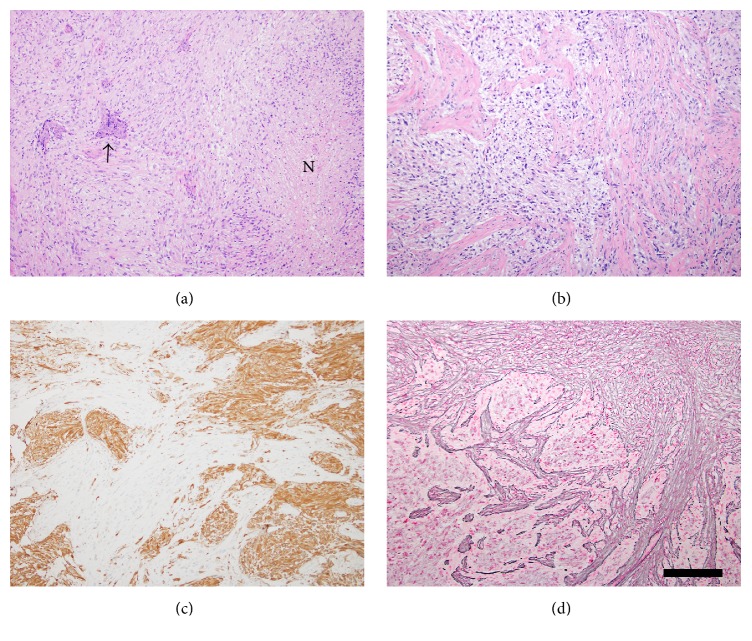
(a) The hematoxylin and eosin-stained histological sections showed an infiltrating glioma containing microvascular proliferation (arrow) and foci of necrosis surrounded by vaguely pseudopalisading tumor cells (“N”), diagnostic of glioblastoma. (b) Other areas showed a malignant mesenchymal component admixed with the glioblastoma. (c) The GFAP stain highlights the glial component and is negative in the mesenchymal component. (d) In contrast, the reticulin stain demonstrates extensive pericellular deposition of collagen fibers in the mesenchymal component only and is negative in the glial component. These findings support the diagnosis of gliosarcoma (all images at 100x magnification; scale bar = 300 *μ*m).

Pathology revealed a malignant neoplasm with variable morphology and extensive infiltration of adjacent fibroadipose and muscle tissue. Gliofibrillary cytoplasmic processes and cerebral parenchymal investment consistent with infiltrative glioma were widely observed, with foci of necrosis surrounded by vaguely pseudopalisading tumor cells (Figure 4(a)). The infiltrative glioma areas were also noted to alternate with mesenchymal-pattern areas of fibrotic stroma comprised of tumor cells with elongated, spindle-shaped nuclei (Figure 4(b)). Other characteristic GB features included positive immunohistochemical staining for glial fibrillary acid protein, which was notably absent from mesenchymal-predominant regions (GFAP, Figure 4(c)). In parallel, reticulin staining showed a dense pericellular deposition pattern in mesenchymal regions and was negative in GFAP-positive areas (Figure 4(d)). Malignant cells were negative for mutant IDH1-R132H protein, with retained ATRX expression and no* IDH1* or* IDH2* mutation detectable by pyrosequencing. Methylguanine-DNA methyltransferase (*MGMT*) promoter methylation was not detected by a methylation-specific PCR-based assay.

## 3. Discussion

Primary GS is an uncommon tumor and in its prototypical form rarely extends beyond the dura in the absence of preceding radiation or craniotomy. Specific invasion of the skull base is exquisitely rare for primary GS, with three prior cases appearing in the literature [7–9], two of which involved both the skull base and either the surrounding parenchyma or extracranial soft tissue [8, 9]. We report the first case of primary intracranial GS with multicompartment invasion of the adjacent parenchyma, skull base, extracranial soft tissues, and orbit.

Literature review for case reports was completed by searching PubMed using keywords “gliosarcoma” together with “extracranial” or “skull base.” Initial search, primary review, and secondary bibliographic review identified 29 publications from 1985 to 2013. Six of these were confirmed cases of GS with involvement of the skull base [7–12]. Four reported secondary invasive GS in the setting of previously resected and radiated primary GB (Schuss et al. 2011, Murphy et al. 1985, Maeda et al. 2010, and Oberndorfer et al. 2013); two cases of primary GS with involvement of multicompartment infiltration were identified (Borota et al. 2006 and Sade et al. 2006). Based on the six confirmed cases of GS involving the skull base and our case report, headache was the most common presenting symptoms (*n* = 6, 86%); proptosis was not previously observed, whereas cranial neuropathies, papilledema, and mass effect were inconsistently reported.

Although the number of studies available for comparison is small, MRI characteristics of GS invading skull base appear to be consistent. They typically demonstrate heterogeneous, peripheral enhancement attributable to frequent tumor hemorrhage and internal necrosis, with a corresponding predominance of peritumoral cytotoxic edema that may obscure clear radiographic differentiation at the tumor-parenchyma interface. Involvement of the infratemporal fossa or sphenoid sinus is common, with each occurring in roughly two-thirds of patients (*n* = 5, 71%). In the setting of primary GB, progression to GS after radiation occurred within 2–6 months. Mortality was high with only one patient surviving beyond 12 months; death is most frequently attributed to metastatic disease, including spread to the lungs and spine [9].

The mechanism of extradural and extracranial extension by GS remains unclear, although the most important barrier to tumor dissemination is thought to be the dura, which plays a role in the containment of glial and other related CNS malignancies [13]. Several candidate mechanisms for invasion of the skull and meninges by astrocytomas were proposed by Kawano et al. [14], who listed three key possibilities: via perivascular or dural slits, along the cranial or spinal nerves, or through direct destruction of the cranial architecture. Still other plausible mechanisms were advanced by Shenoy and Raja, who theorized dural necrosis resulting from a combination of disrupted blood supply and bone invasion [15].

Although GS treatment falls within the broader paradigm of GB management, several studies have reported that temozolomide does not significantly impact overall survival [16, 17]. One recent retrospective study of 75 patients with GS has reported that neither temozolomide-based chemoradiation nor adjuvant chemotherapy was superior to radiotherapy alone [18]. Correspondingly, they recommended surgery with adjuvant radiation at a minimum dose of 54 Gy as standard-of-care therapy for GS. In our presented case, 76 Gy of adjuvant radiation was administered.

Sequencing and comparative genomic hybridization studies have helped elucidate differences between GB and GS, although their results are limited by the lack of randomized studies or molecular data, both of which are attributable to the rarity of GS [4]. Notwithstanding, limited but stepwise discoveries are optimizing treatment protocols, as in the specific example of GS frequently lacking the overexpression of epidermal growth factor receptor (EGFR) seen in IDH-wildtype GB, which challenges the utility of anti-EGFR modalities in GS treatment [19]. Still other studies of the molecular alterations in GS have found a high incidence of TP53 mutations, as well as rare EGFR and IDH mutations [20, 21]. Further study of the molecular mechanisms underlying GS development and spread is required to better understand the natural history and optimal treatment of these lethal tumors.

## 4. Conclusion

To our knowledge, this is the first reported case of a primary intracranial GS with direct invasion of the skull base, brain parenchyma, extracranial compartment, and orbit. This case report illustrates how rapid and aggressive the natural history of GS can be. Further this case report adds to the radiographic differential of a mass involving the soft tissues, bone, and intra-axial compartments beyond aggressive meningioma, metastasis, primary bone neoplasm, or sarcoma. Although GS is rare and similar to GB, the higher mortality of GS and notable molecular differences between GS and GB urge the need for specialized treatment modalities beyond mild alterations of standard GB treatment.

## Abbreviations

GB: Glioblastoma
GS: Gliosarcoma.
